# A critical review on various feedstocks as sustainable substrates for biosurfactants production: a way towards cleaner production

**DOI:** 10.1186/s12934-021-01613-3

**Published:** 2021-06-26

**Authors:** Swayansu Sabyasachi Mohanty, Yamini Koul, Sunita Varjani, Ashok Pandey, Huu Hao Ngo, Jo-Shu Chang, Jonathan W. C. Wong, Xuan-Thanh Bui

**Affiliations:** 1 Gujarat Pollution Control Board, Gandhinagar, Gujarat 382 010 India; 2 Central University of Gujarat, Gandhinagar, Gujarat 382030 India; 3 CSIR-Indian Institute of Toxicology Research, Lucknow, 226 001 India; 4 Centre for Technology in Water and Wastewater, School of Civil and Environmental Engineering, University of Technology Sydney, Sydney, NSW 2007 Australia; 5 Department of Chemical Engineering, National Cheng Kung University, Tainan, Taiwan; 6 Institute of Bioresource and Agriculture, Hong Kong Baptist University, Kowloon Tong, Hong Kong; 7 Faculty of Environment and Natural Resources, Ho Chi Minh City University of Technology (HCMUT), Ho Chi Minh City, 700000 Vietnam; 8 Key Laboratory of Advanced Waste Treatment Technology, Vietnam National University Ho Chi Minh (VNU-HCM), Linh Trung Ward, Thu Duc District, Ho Chi Minh City, 700000 Vietnam

**Keywords:** Biosurfactant, Cleaner production, Agro-industrial waste, Municipal solid waste

## Abstract

The quest for a chemical surfactant substitute has been fuelled by increased environmental awareness. The benefits that biosurfactants present like biodegradability, and biocompatibility over their chemical and synthetic counterparts has contributed immensely to their popularity and use in various industries such as petrochemicals, mining, metallurgy, agrochemicals, fertilizers, beverages, cosmetics, etc. With the growing demand for biosurfactants, researchers are looking for low-cost waste materials to use them as substrates, which will lower the manufacturing costs while providing waste management services as an add-on benefit. The use of low-cost substrates will significantly reduce the cost of producing biosurfactants. This paper discusses the use of various feedstocks in the production of biosurfactants, which not only reduces the cost of waste treatment but also provides an opportunity to profit from the sale of the biosurfactant. Furthermore, it includes state-of-the-art information about employing municipal solid waste as a sustainable feedstock for biosurfactant production, which has not been simultaneously covered in many published literatures on biosurfactant production from different feedstocks. It also addresses the myriad of other issues associated with the processing of biosurfactants, as well as the methods used to address these issues and perspectives, which will move society towards cleaner production.

## Introduction

Surfactants are a diverse group of synthetic and biological compounds that all have a common tension-active property and are used in nearly all our everyday routine tasks [1]. The bulk of these surfactants are made from petroleum and are chemically synthesized [2]. In contrast, due to environmental concerns about chemical surfactants, the recent push for environmentally friendly technology has increased the use of microbial surfactants [3, 4].

**Fig. 1:**
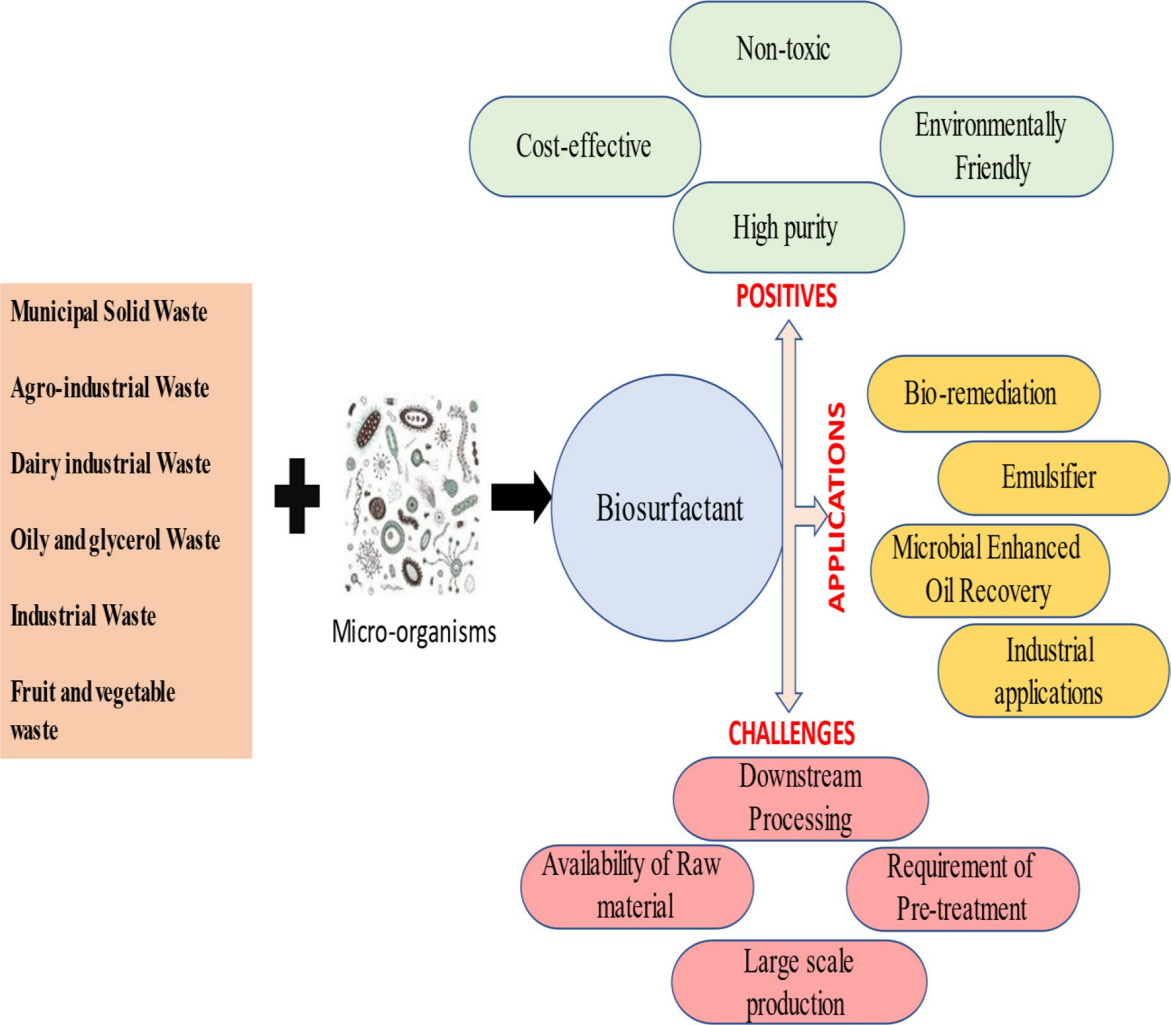
Biosurfactant: feedstocks, benefits, drawbacks, and applications

Biosurfactants are gaining popularity due to their remarkable advantages over synthetic ones—low toxicity, biodegradability, efficacy over a broad pH and temperature range are just a few to name [5–7]. As a result, it is now used in a wide range of fields, including environmental [8–10], food [11, 12], remediation [13], biomedical [14–16], and a variety of other commercial applications [17–20]. The different feedstocks used, benefits, applications, and drawbacks of biosurfactants are depicted in Fig. 1.

**Fig. 2:**
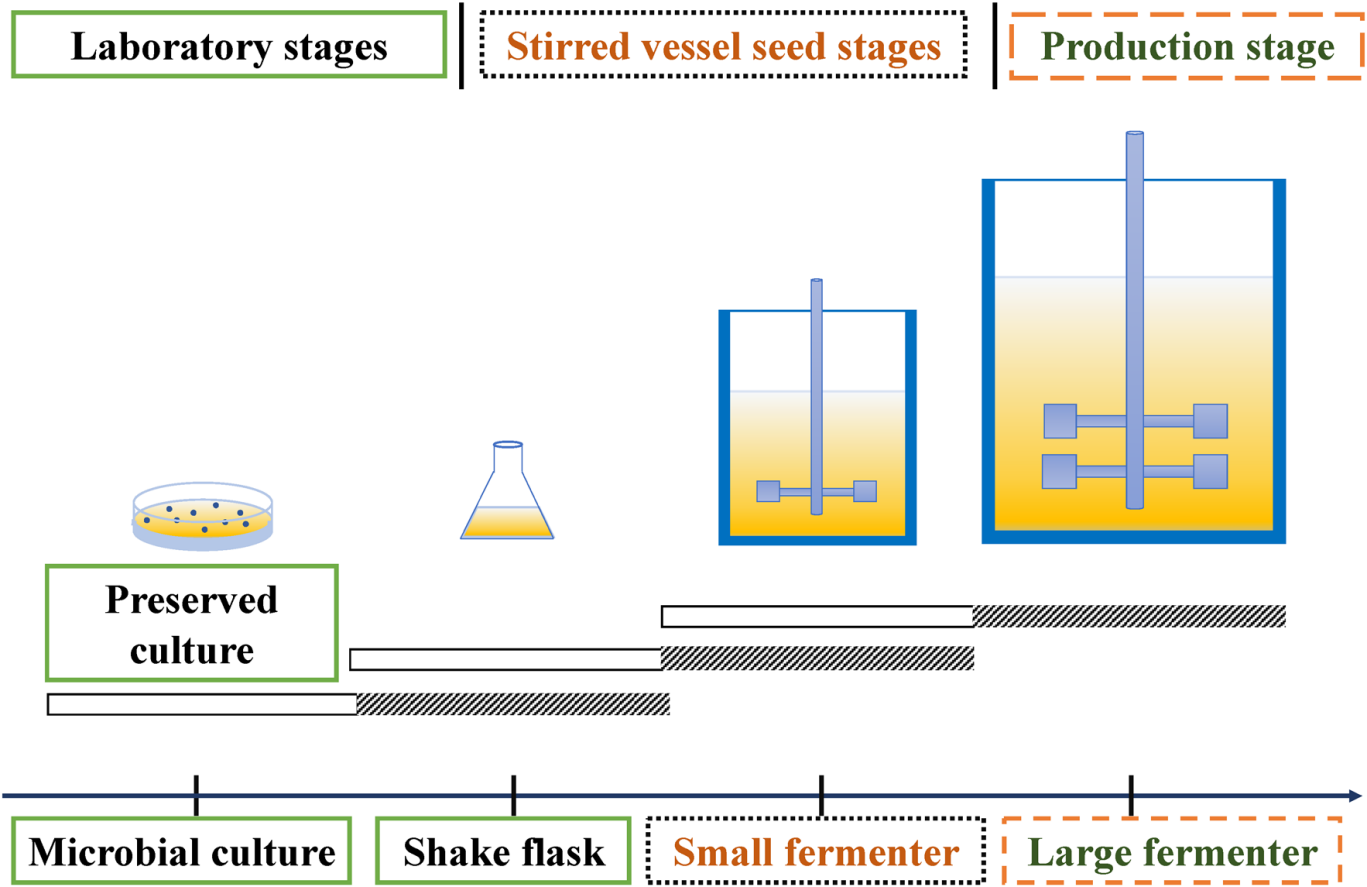
Schematics of scale-up process for biosurfactant production

These biosurfactants are produced under a wide range of growth and environmental conditions, and they are known to be primarily involved in enhancing the solubility and availability of different water-immiscible substrates. The members of the genera *Pseudomonas*, *Bacillus*, *Rhodococcus*, and *Candida* are predominantly used for the production of various biosurfactants [21]. A typical biosurfactant is made up of two components: a hydrophilic component and a hydrophobic component [22–24]. The molecular weight, physicochemical properties, and mode of action of these compounds are being used to classify them. According to these combinations, there are low or high molecular weight biosurfactants [25]. The low-molecular-weight biosurfactants lower the surface and interfacial tensions whereas high-molecular-weight biosurfactants, also recognized as bio emulsifiers, are better at stabilizing oil-in-water emulsions [26–28]. Among all currently recognised biosurfactants, Rhamnolipids, which is one of the glycolipids, has the greatest potential to become the next generation of biosurfactants [29]. *Pseudomonas* species is mainly used to produce these important groups of microbial surfactants around the world [10, 13]. To meet the high demand for biosurfactants, the production process has been scaled up and is now being used successfully at various biosurfactant production units. Figure 2 shows a schematic representation of the upscaling of the biosurfactant production.

According to a recent study, the global Biosurfactants market is anticipated to grow at a CAGR of 0.8 percent from US$ 1.3754 billion in 2020 to US$ 1.4427 billion in 2026 [30]. However, despite the high market demand, the cost of producing a biosurfactant is higher than that of synthetic ones [31]. Too much foaming while batch processing, lower yield, availability of affordable raw materials, expenses involved with downstream processing and purification, are still some challenges in biosurfactant production at the industrial scale [32–34]. As a result, the success of biosurfactant production hinges on the creation of less expensive processes, particularly in the aspect of substrates, which account for 10–30% of total production costs [35, 36]. To address this problem, processes could be linked with the use of waste as substrates which would minimize pollution while balancing overall costs [37, 38]. This method lowers the cost of waste treatment while also providing the opportunity to benefit from the selling of the biosurfactant. Industrial, agricultural, food waste, and other low-cost substrates may be used to improve this situation [39–43]. Furthermore, techniques such as Response Surface Methodology (RSM) and various statistical approaches have been successfully used in several studies to reduce the cost and time consumed for media optimization in order to optimise biosurfactant manufacturing operations [31]. Many studies have also used engineering techniques to increase output and reduce the downstream production costs.

The current review provides an up-to-date study on techniques available for biotransformation of sustainable substrates into value-added products such as biosurfactant, as well as its significant contribution to the generation of a circular bioeconomy. Furthermore, the paper addresses the use of municipal solid waste as a substrate for biosurfactant production, which has yet to be addressed in most studies. It also addresses the myriad of other issues associated with the processing of biosurfactants, as well as the methods used to address these issues. The review also discusses the pre-treatment techniques used, as well as the research needs and prospects for using sustainable substrate for biosurfactant production.

## Need for waste stream derived cleaner production

**Fig. 3:**
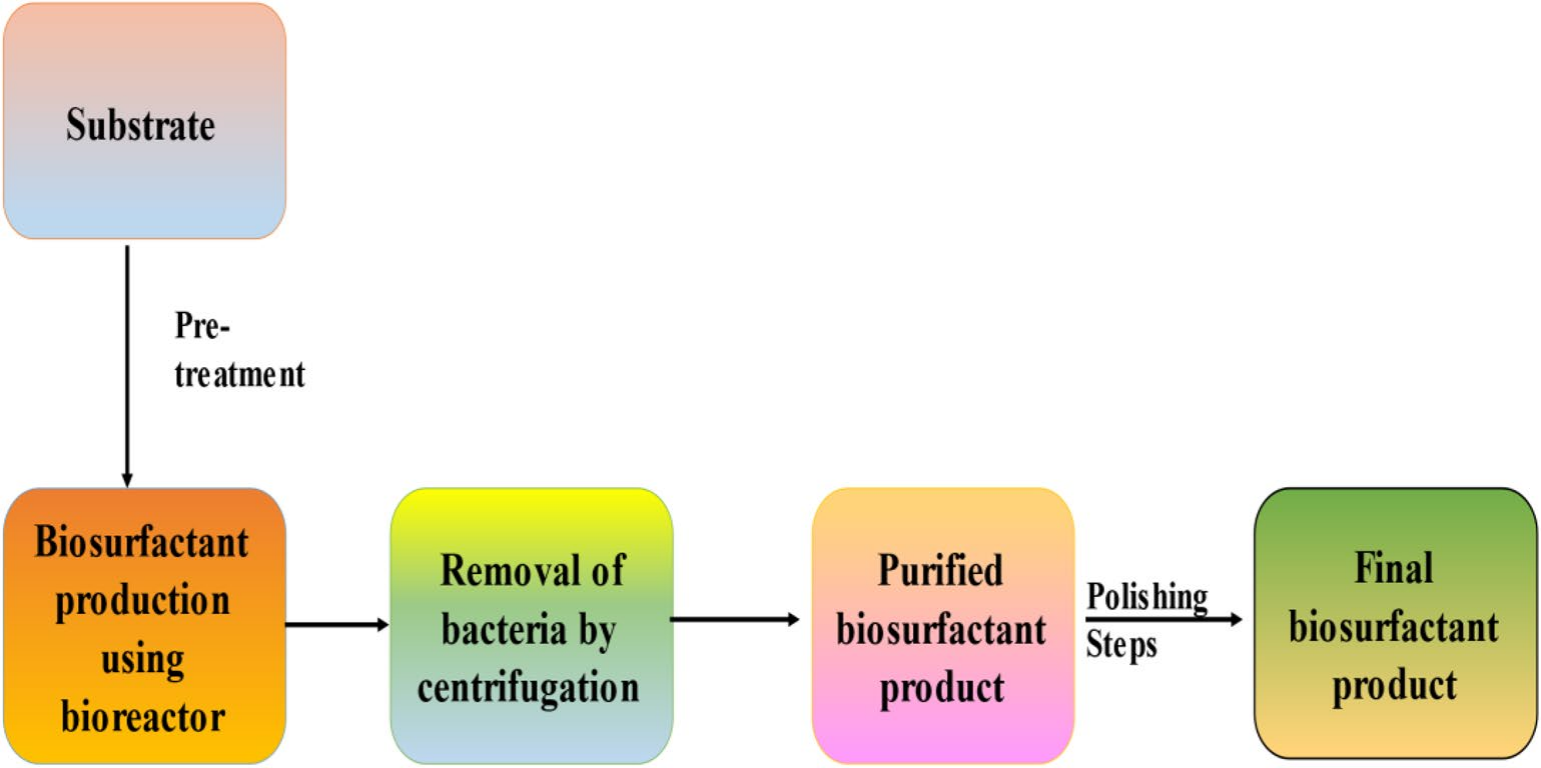
Graphical representation on biosurfactant production

The concept of using the waste stream for the production of useful material is based on the pursuit of a viable and sustainable method for transforming waste into a jewel [44]. It closes the open loop leaving less waste unutilized thereby, causing less harm to the environment and health [45–47]. This circular loop of waste utilization is the best waste reduction and resource recovery option [48–52]. Because, the use of the waste stream for biosurfactant production has many benefits, including lower processing costs, widespread availability of many less expensive/renewable substrates, and most importantly, the product becomes more environmentally friendly while maintaining its basic functional properties [53, 54]. Figure 3 depicts the biosurfactant production schematics.

According to the United Nations Environment Programme [55], approximately 93.1 crore tonnes of food waste were produced in 2019, with 61% coming from residences, 26% from food service, and 13% from retail; implying that 17% of total global food production could be squandered. Similarly, in India, the annual wastage of food is projected to be 68,760,163 tonnes. Such wastage of food has social and economic consequences along with significant environmental consequences such as contributing to greenhouse gas emissions and ecosystem degradation through land conversion and pollution etc. [56, 57]. Hence, utilizing these wastes for the production of bioresource will not solve the existing problem like wastage and pollution but will also lead to the production of more useful resources [58–60].

## Suitable substrates for the production of biosurfactant

### Biosurfactant production employing agro-industrial waste and its by-products

Over the last 10 years, there has been a surge in the need for cost-cutting materials which could act as the substrates for biosurfactant production. A variety of renewable and cheap industry-based wastes are being studied and examined for their potential as substrates for biosurfactant production. Among these are the agro-industrial wastes and food residues [61, 62]. This approach of using waste as the substrate is not only helpful in increasing the profitability of the process but also aids in the effective management of the waste that is being generated at an unprecedented pace [63, 64]. However, along with the cost efficiency, factors like the stability, form, and amount to be used, etc., are also taken into consideration while choosing a substrate for biosurfactant production [65, 66].

Microbial species like *Halobacteriaceae archaeon*, *Pseudomonas aeruginosa*, *Bacillus pumilis*, *Bacillus licheniformis*, *Candida tropicalis*, etc. are used for the biosurfactant production from agro-industrial waste like date molasses, cassava waste, orange peel, corn steep liquor, sugarcane bagasse, etc. [67, 68]. The production of biosurfactants from agro-industrial waste has several advantages, including increased cost efficiency, large-scale substrate production, increased availability of a broader spectrum of substrates, intact functional properties, environmentally friendly products, and non-toxic to associated microorganisms [1, 3, 69].

#### Production of biosurfactants using fruit and vegetable waste

Fruits and vegetables processed for their juices and other products produce a lot of waste like peels of apples, banana, orange, carrots, etc. which have the potential to be used as the substrates for biosurfactant production [23, 70]. Studies have been done to evaluate the production of biosurfactants using cashew apple juice using *Acinetobacter calcocetius* [61, 71]. The evaluation showed a reduction in the surface tension up to 17%. Similar studies done on *Pseudomonas aeruginosa* showed that the highest reduction was about 29.5 mN/m when *Pseudomonas aeruginosa* produces rhamnolipids in a mineral medium nourished with cashew apple juice [72].

Another potent waste product that has been taken into consideration for use as a substrate is banana peel, which is the primary by-product of the production and processing of bananas. Banana peel has been used as the only carbon source used in the synthesis of lipopeptides using *Halobacteriaceae archaeon* [73]. Along with banana peels, carrot peel waste, lime peelings and orange peels have been used to produce rhamnolipids using *Pseudomonas aeruginosa* [74, 75] *.*

#### Production of biosurfactants using starch-rich waste

Extraction of starch from rice, wheat, cassava, potato, and other crops generates a vast amount of wastewater that is high in starch and husks and thus can be used as a feedstock to produce various products including biosurfactants [76, 77]. *Bacillus subtilis* was used to evaluate potato substrate as an unconventional carbon source for surfactant production. It was also used to produce biosurfactants using cassava wastewater. Furthermore, utilising soybean flour and rice straw as substrate, *Bacillus amyloliquefaciens* was used to produce lipopeptides [78].

### Biosurfactant production employing industrial waste

The industry-based need for surfactants is on a constant rise. Biosurfactants are used in the bioremediation of hydrocarbon and petroleum polluted soil and groundwater, as well as to degrade other persistent harmful compounds. Biosurfactants are gaining lots of interest in recent times because of their natural origin, low toxicity, and environment-friendly properties. A wide variety of substrates can be used for the production of biosurfactants like dairy-production waste, waste from fruit juice processing industries, oil processing industry-derived waste, etc. [39].

Oil processing and production at large scale generates a great amount of waste which are of varied types like soap stock, marine oils, lard, tallow, and/or free fatty acids from the extraction of oil from seeds. The disposal of such huge quantity of waste is an ongoing concern and therefore their use as a substrate for the biosurfactant is garnering a lot of attention from researchers around the globe [79, 80]. Yeasts have been employed to produce biosurfactants using industrial wastes like oils and free fatty acids due to their ability to produce emulsifiers. Among the yeast species, the species *Candida* has been most widely employed to produce biosurfactants using oil residues generated from oil processing industries. Other microbes employed to produce biosurfactants using industrial wastes are *Corynebacterium aquaticum*, *Candida bombicola*, *Candida utilise* etc. [81, 82].

### Biosurfactant production employing lignocellulosic waste

Lignocellulose is an abundant organic carbon source that is widely available. The cellulose is primarily derived from the plants that are potentially grown for their cellulosic content. The tendency of a microorganism to produce biosurfactant using lignocellulosic media as a substrate was studied on *Lactobacillus pentosus*, using hydrolysed distilled grape marc which consists of 10.8% cellulose, 11.2% hemicellulose, and about 51% lignin. The growth media was supplemented by yeast extract and corn steep liquor. An intercellular biosurfactant production of about 4.8 mg/L was reported for this experimental setup. Similarly, the bacterial strain of *Bacillus tequilensis*, which was isolated from Mexican brines were used to produce both intracellular and extracellular biosurfactant [83–85].

The wide array of studies in context to biosurfactant production using lignocellulosic substrate has highlighted that the possibility of producing a variety of biosurfactants using different carbon sources, but some strains can increase the industrial application potential of the microbial strains and biosurfactants. The lignocellulosic substrates are therefore an excellent cost-efficient carbon source for biosurfactant production. However, the cost of producing biosurfactants rises due to the pre-treatment processes required to ensure that the lignocellulosic residues are available for the microbes to act on. Pre-treatment of lignocellulosic residue includes particle size reduction, pre-hydrolysis, chemical/enzymatic hydrolysis, and drying. The microbe strains that use lignocellulosic residues are *Lactobacillus paracasei*, *Starmerella bombicola*, *C. bombicola*, *Cutaneotrichosporon mucoides* [86, 87].

### Biosurfactant production employing oily and glycerol-based waste and other substrates

Surfactants are a critical class of chemicals with numerous industrial applications. Thus, the production of biosurfactants from natural substrates not only aids in the management of industry-derived waste, but also in the production of surfactants that are more environmentally friendly and less toxic [88]. Aside from the substrates mentioned above for the production of biosurfactants, some other substrates that can be used for the production of biosurfactants are as follows:

#### Production of biosurfactants using frying oil wastes

Many of the agricultural products are processed to produce different food commodities. The residues of this food processing in industries are frequently used frying oils, the nutritional values of which vary depending on the products fried in the oils and the number of times they were reused for frying. The used frying oil has a higher concentration of polar hydrocarbons than the fresh oil and its major constituents are primarily monoglycerides, diglycerides, and triglycerides along with some proportion of free fatty acids [89, 90].

Studies have been conducted to investigate the use of waste olive oil and sunflower oil to produce rhamnolipids using *Pseudomonas aeruginosa* [64]. Similar studies revealed that the *Candida bombicola* strain was capable of producing sophorolipids from oil waste. Other microbial species that have successfully demonstrated the production of biosurfactants using used frying oil as the substrate medium are *Bacillus subtilis*, *Bacillus stratosphericus*, *Streptomyces*, *Pseudomonas cepacia*, and *Mucor circinelloides* [91, 92].

#### Production of biosurfactants using wastes from vegetable oil processing and its by-products

The processing of vegetable oils generates a large amount of waste which consists of a high concentration of fats, oils, and other associated compounds. These residues are potent contaminants that can lead to both soil and water pollution. Their potency to act as pollutants can be attributed to the low degradability of the lipids compounds that they contain [93]. However, investigative studies have shown that microbial species like *Pseudomonas* can produce rhamnolipids by using olive oil mill effluents and soybean oil refinery waste as a substrate. In similar studies, *Candida sphaerica* successfully produced biosurfactants using groundnut oil refinery waste. Other microbial strains that have been successfully employed for the production of biosurfactants using oil processing wastes are *Bacillus subtilis*, *Starmerella bombicola*, *Trametes versicolor*, etc. [94, 95].

#### Production of biosurfactants using dairy industrial wastes

Dairy industry produces a significant amount of waste in the form of by-products such as whey, buttermilk, and other derivatives [96]. The Biochemical Oxygen Demand (BOD) of the wastes generated by the dairy industry particularly that of whey is significantly high. The disposal of waste is a problem particularly for those countries that predominantly rely upon the dairy economy. Reportedly, only up to 50% of the total waste generated from the dairy industry is recycled into other useful products like animal feed, while the remaining portion is considered waste [97].

Using a two-stage cultivation process, [98] successfully demonstrated the production of increased concentrations of sophorolipids of about 422 g/L. During the first step, *Cryptococcus curvatus* was cultivated in the deproteinized lactose-rich whey concentrate. This was followed by high-pressure homogenization of the biomass generated in the first step to produce a crude cell extract containing a single cell oil. This oil is then utilized by *Candida bombicola* for producing sophorolipids [99].

#### Production of biosurfactants using sugar industrial waste

Molasses is the main by-product of sugar industries involved in both sugar beet and sugar cane industries. Molasses has garnered a lot of popularity as the substrate used for biosurfactant production. The popularity owes to the fact that molasses is a much low-priced source of sugar than other sources and has an adequate number of other compounds and vitamins [100]. *Bacillus subtilis* and *Pseudomonas aeruginosa* bacterial strains have been successfully used in over two decades of research to produce biosurfactants using molasses as a substrate. The molasses-derived biosurfactant exhibited good surface activity and a high emulsification index, indicating its possible application in microbial enhanced oil recovery [38, 61].

## Pre-treatment of substrates for the production of biosurfactants

The use of biologically derived substrates to produce more environment-friendly biosurfactants has been a widely researched area around the globe in recent the past. However, a series of pre-treatment is recommended for a wide variety of substrates varying from oil-based substrates, starch substrates, lignocellulosic substrates etc. Pre-treatment of the substrates such as lignin aids in the production of biosurfactants by de-crystallizing the cellulosic structure, decreasing the content in the substrate, and increasing the surface area to enhance the enzymatic activity of the enzymes produced by the microbes using the substrate. The pre-treatment of this substrate biomass makes more sugar available for the microorganism to act upon [101, 102].

The primary step of pre-treatment is the size reduction of the substrate feedstock to ensure better utilization of the feedstock by the microorganisms. The substrate is reduced in size using equipment like hammer mill, tub grinder, etc. The particle size reduction helps to increase total surface area, pore size, and contact points for an enzyme to act upon the substrate. The particle size reduction is followed by the pre-hydrolysis treatment which is either done by using liquid ammonia or by using ultrasonication. The use of liquid ammonia has proved to be one of the most efficient delignification techniques and has been popularly been employed in the pre-treatment of lignocellulosic substrates like corn stover. The technique of ultrasonication is a relatively less explored technique for pre-treatment. It is a physical pre-treatment method. It is believed to have the potential to change the substrate structure by de-crystallizing the cellulosic part while retaining a significant amount of substrate and polysaccharide [86, 103].

The substrate is then chemically/enzymatically hydrolyzed. The chemical hydrolysis of substrates can be classified into two types—acid hydrolysis and alkaline hydrolysis. The acid hydrolysis employs inorganic acids like HCl and H_2_SO_4_ in either concentrated or diluted forms to treat the substrates for biosurfactant production. Although the concentrated form of acid yields better results, it makes the process more expensive. Therefore, dilute-acid hydrolysis has been successfully employed for the pre-treatment of substrates. Similarly, alkalis like potassium, calcium, sodium, and ammonium hydroxides have been used for pre-treatment and unlike acid hydrolysis, they cause less sugar degradation. Apart from the chemical alternatives, enzymes derived biologically can also be employed for hydrolysis treatment. Enzymes like β-glucosidase have been successfully employed in the hydrolysis treatment of bagasse and other substrates [36, 104].

**Fig. 4:**
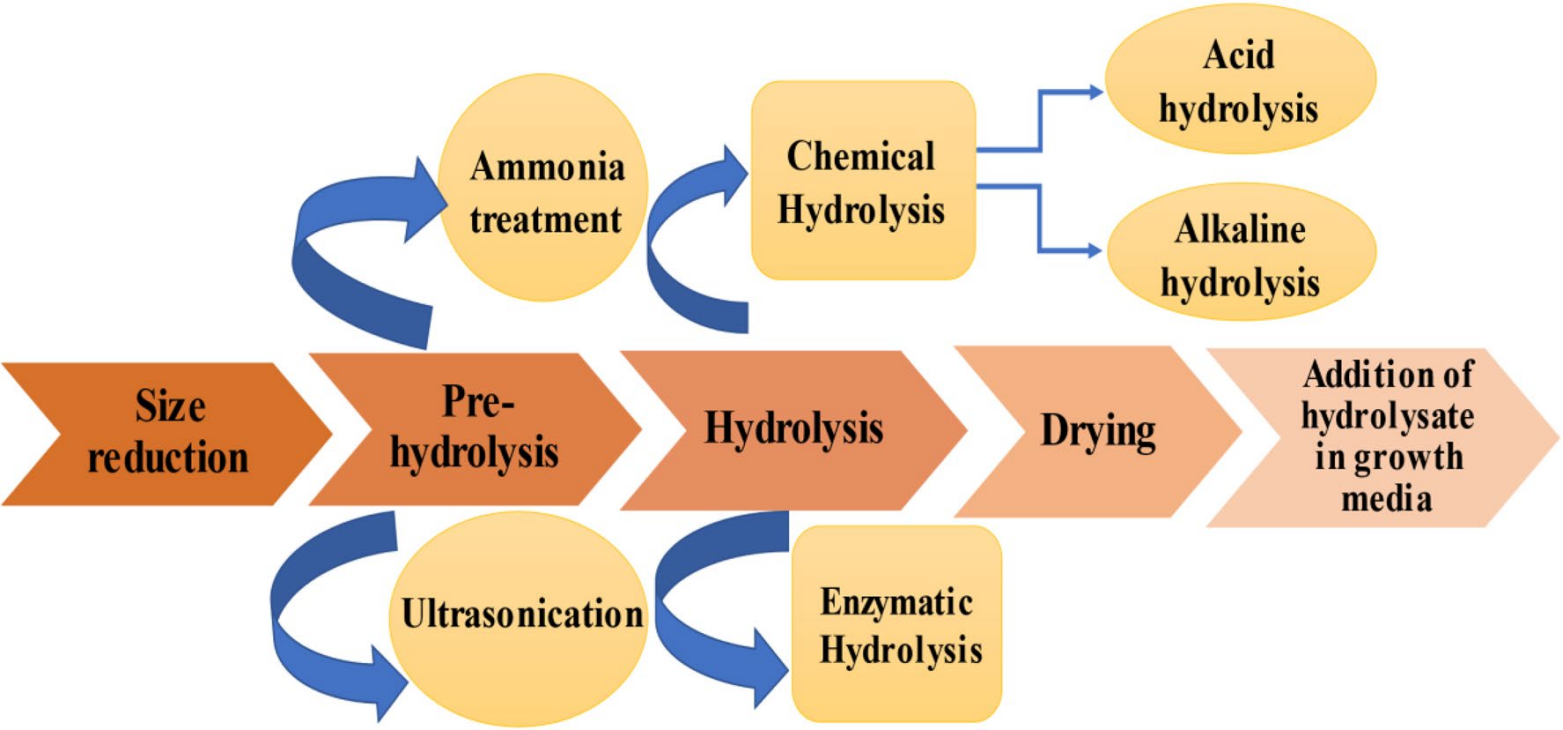
A schematic image for pre-treatment technology used for biosurfactant production

The final step of the pre-treatment process is drying of the substrates (hydrolysates). The substrate is then incorporated into the microbial growth media wherein the substrates are used by the microbes as the primary source of sugar for growth followed by the production of biosurfactant in the form of metabolites [105–107]. Depending on the substrate to be treated, the pre-treatment steps can be used individually or in the sequence outlined above. The pre-treatment process is pivotal for obtaining a high monosaccharide content and limiting the amount of inhibitory compounds in the hydrolysates, which affects the yield, efficiency, and cost of the biosurfactant production process [108]. Figure 4 depicts a schematic image for pre-treatment technology used for biosurfactant production.

## Strategies for biosurfactant production using municipal solid waste

Biosurfactants can be made from a variety of low-cost raw materials that are readily available. The carbon source could be hydrocarbons, carbohydrates or lipids, which could be used individually or in combination. Several low-cost waste materials have been investigated as biosurfactant substrates over the last decade, resulting in a cost-effective strategy [109–112]. Because of the high C/N concentration in MSW, producing biosurfactants from it is a viable and advantageous option [109, 113].

The generation of municipal solid waste has also accelerated as a result of urbanization, a growing population, a thriving economy, and a rapid increase in the living standards of people [114–116]. This poses a significant obstacle to the environment and long-term development [117, 118]. Thus, adopting an effective Municipal Solid Waste Management (MSWM) plan such as bioconversion of waste materials is regarded as critical for the coming years because it solves the problem of environmental pollution while also providing an option for resource recovery [48, 119–122].

According to the United Nations Environment Programme [55], approximately 93.1 crore tonnes of food waste were produced in 2019, with 61% coming from residences, 26% from food service, and 13% from retail; implying that 17% of total global food production could be squandered. Avoiding food waste is therefore critical for all those involved in food processing, distribution, and marketing [123]. The proper management of these products at the end of their lifecycle is important in effort to prevent the environmental and social impacts caused by untreated, decomposing food. In addition to wasting energy used to produce food that is not eaten, poorly managed food waste impacts our atmosphere by releasing greenhouse gases during decomposition, pollutes waterways by nutrient and leachate runoff, and can be a disease vector [49].

Food waste is a plentiful and potentially useful source of feedstock. Many studies have shown that the MSW stream in India, contains the highest proportion of biodegradable and recyclable waste, with food waste dominating the organic waste composition [27, 28]. It primarily consists of residual food debris, vegetable waste, leaves, and decaying vegetables. Even so, internationally, poor and middle-income countries produce the majority of MSW with a higher organic/biodegradable content [124]. Valorization of food waste is becoming increasingly critical for achieving Sustainable development goals (SDG’s) like food security, environmental protection, etc. [113, 125]. Since food waste contains a high concentration of organic content, traditional disposal and incineration methods can endanger the environment and human health by releasing toxic gases [126, 127]. As a result, using these wastes as a substrate for biosurfactant production offers a renewable method of valuing. Food waste in MSW can be manually separated and used as a substrate for biosurfactant production, lowering production costs and emissions.

Once segregated the food waste can be utilized for biosurfactant production. Many pieces of research have previously been done where food waste has been used as a low-cost substrate for Biosurfactant production. For example, Kitchen waste oil is high in protein and moisture which promotes microbial development. In an experiment [91] successfully used *Pseudomonas aeruginosa*, isolated from kitchen waste oil, and used it as a fermentation substrate over glucose, glycerol, molasses, and rapeseed oil for the production of biosurfactants. They discovered that the process was optimized at a pH of 8.0 and a nitrogen source concentration of 2.0 g/L, and they received a biosurfactant made from a mixture of six rhamnolipid. Table 1 shows the production of different types of biosurfactant using various substrates.

**Table 1:** Biosurfactant production from various feedstocks and microorganism used

Feedstock	Microorganism used	Type of biosurfactant	Applications	References
Fruit and vegetable waste	*Halobacteriaceae archaeon* *Pseudomonas aeruginosa* *Bacillus subtilis*	Lipopeptides Rhamnolipids Surfactin	Can be used in bioremediation of oil contaminated sites, cosmetic industry, and pharmaceutical industries	Kumar et al. [75]; Paraszkiewicz et al. [128] Varjani and Upasani [10]
Starch rich waste	*Bacillus subtilis* *Pseudomonas aeruginosa*	Surfactin Rhamnolipids	Reported use in oil recovery, environmental protection, and pharmaceutical industries	de Andrade et al. [78] Sharma et al. [9]
Frying oil waste	*Candida bombicola* *Pseudomonas aeruginosa*	Sophorolipids Rhamnolipids	Potential in environmental applications, bioremediation of oil and hydrocarbon contaminated sites	Hasanizadeh et al. [129]; Ozdal et al. [92] Santos et al. [130]
Oil processing waste and by-products	*Pseudomonas aeruginosa* *Bacillus subtilis*	Rhamnolipids Lipopeptides	Remediation of oil and hydrocarbon contaminated sites	Lourenço et al. [131] Jadhav et al. [93] Varjani et al. [64]
Lignocellulosic wastes	*Candida bombicola* *Starmerella bombicola*	Sophorolipids	Potential in environmental applications	Chen et al. [91] Marcelino et al. [100] Kaur et al. [132]
Kitchen organic waste	*Pseudomonas aeruginosa* *Starmerella bombicola*	Rhamnolipid Sophorolipid	Reported to be used in development of waste-based bio-processes	Li et al. [133]; Zhao et al. [62] Liu et al. [134]; Marcelino et al. [100]

Similarly in another experiment, *Starmerella bombicola ATCC 22214* was used by [132] for enzymatic hydrolysis of mixed food waste. They obtained a hydrolysate containing 99.1 g/L glucose and 2.4 g/L FAN after completing the food waste hydrolysis. Besides, the C/N ratio of the mixed food waste hydrolysate used in the study was discovered to be 41. As a result of the presence of a suitable nutrient balance in food waste hydrolysate, they concluded that it was the best waste feedstock for sophorolipids production. This suggests that food waste could be useful in the development of waste-based bio-processes for the production of biosurfactants.

Furthermore, fruit and vegetable peelings, which are also a component of the biodegradable fraction of MSW, can be used to create biosurfactant [135] used submerged fermentation to generate rhamnolipid biosurfactant from *Pseudomonas aeruginosa MTCC 2297* using a variety of low-cost waste materials like orange peelings, coconut oil cake, lime peelings, carrot peel waste, and banana waste, among others. Orange peel was discovered to be the best substrate, producing 9.18 g/L rhamnolipid biosurfactant with a surface tension reduction of up to 31.3 mN/m.

## Road blocks and future perspectives

The concept of using MSW as a substrate for biosurfactant production is exciting, but there are still many obstacles to address before it can be scaled up. There is a scarcity of research to back up the claims that MSW can act as a primary sustainable substrate, thereby reducing biosurfactant production cost and resulting in an increase in business profit. Furthermore, the profitability of these processes is influenced by the composition of waste, which varies from location to location due to the economic class of the people living there, the climate of the place, and so on [136, 137]. In addition to this the waste segregation techniques followed, transportation costs associated with it, and the energy expenditures that occur during the process; make it more challenging [138].

Similarly, the choice of substrate used is also crucial in determining the yield and efficiency of the end product obtained. Basic considerations particularly concerning substrates like the agro-industrial substrates must consider factors like availability of raw material, the cost of transportation of raw material, and the pre-treatment required before the production process. Efforts should be made to reduce or if possible, eliminate the pre-treatment steps to reduce the cost of production. At the same time, research efforts should be made to find more favourable microbes that can thrive and use the available agro-industrial waste in the less treated form and produce a higher yield of biosurfactants. In this context, recombinant microbes can be employed to treat the available substrate and get the desired surfactant in a higher yield [139].

Downstream processing is plays major role in all the expenses of every biotechnology product [140, 141]. A wide range of analytical techniques has been used to isolate, purify, and classify various biosurfactants. A substantial amount of research has been conducted to increase the productivity and yield of biosurfactants in the upstream production process [142, 143], but a comprehensive study on downstream purification is still lacking. These challenges are opportunities for research groups that should be thoroughly examined and addressed.

## Conclusions

Surfactants produced from renewable raw materials such as waste are increasingly finding their way onto the market chain. The key impediment to biosurfactant commercialization is the high cost of large-scale processing. To address the challenge and compete with synthetic surfactants, low-cost substrate, efficient microorganisms, and appropriate engineering techniques should be used for biosurfactant production. It can be aided by improved production conditions created using less expensive renewable substrates like molasses obtained from the sugar industry, vegetables, and frying oils, dairy waste, agro-industrial wastes, food waste and municipal solid waste, etc. However, the cost of pre-treatment, downstream processing, and, most importantly, large-scale production in order to meet market demand remains a barrier in biosurfactant production. As a result, the true value of these processes can only be verified when these studies are successfully applied to a commercially competitive system. The study of these renewable substrates and processes can help researchers and academicians design better experimental setups to refine existing processes and meet the demands of commercial production systems. Waste management is a major challenge in today’s world, and using waste materials as substrates can solve both the problem of waste disposal and its long-term adverse environmental impacts.

## Data Availability

All data and material used for preparing the manuscript appear in the submitted article.
